# Exergames as a rehabilitation tool to enhance the upper limbs functionality and performance in chronic stroke survivors: a preliminary study

**DOI:** 10.3389/fneur.2024.1347755

**Published:** 2024-02-08

**Authors:** Luca Vismara, Claudia Ferraris, Gianluca Amprimo, Giuseppe Pettiti, Francesca Buffone, Andrea Gianmaria Tarantino, Alessandro Mauro, Lorenzo Priano

**Affiliations:** ^1^Division of Neurology and Neurorehabilitation, Istituto Auxologico Italiano IRCCS, S. Giuseppe Hospital, Piancavallo, Italy; ^2^Institute of Electronics, Information Engineering and Telecommunication, National Research Council, Turin, Italy; ^3^Department of Control and Computer Engineering, Politecnico di Torino, Turin, Italy; ^4^Division of Paediatric, Manima Non-Profit Organization Social Assistance and Healthcare, Milan, Italy; ^5^Principles and Practice of Clinical Research, Harvard T.H. Chan School of Public Health–ECPE, Boston, MA, United States; ^6^Department of Neurosciences “Rita Levi Montalcini”, University of Turin, Turin, Italy

**Keywords:** stroke, rehabilitation, exergames, RGB-D camera, upper limb mobility, gait analysis, arm swing asymmetry

## Abstract

**Introduction:**

Post-stroke hemiplegia commonly occurs in stroke survivors, negatively impacting the quality of life. Despite the benefits of initial specific post-acute treatments at the hospitals, motor functions, and physical mobility need to be constantly stimulated to avoid regression and subsequent hospitalizations for further rehabilitation treatments.

**Method:**

This preliminary study proposes using gamified tasks in a virtual environment to stimulate and maintain upper limb mobility through a single RGB-D camera-based vision system (using Microsoft Azure Kinect DK). This solution is suitable for easy deployment and use in home environments. A cohort of 10 post-stroke subjects attended a 2-week gaming protocol consisting of Lateral Weightlifting (LWL) and Frontal Weightlifting (FWL) gamified tasks and gait as the instrumental evaluation task.

**Results and discussion:**

Despite its short duration, there were statistically significant results (*p* < 0.05) between the baseline (T0) and the end of the protocol (TF) for Berg Balance Scale and Time Up-and-Go (9.8 and −12.3%, respectively). LWL and FWL showed significant results for unilateral executions: rate in FWL had an overall improvement of 38.5% (*p* < 0.001) and 34.9% (*p* < 0.01) for the paretic and non-paretic arm, respectively; similarly, rate in LWL improved by 19.9% (*p* < 0.05) for the paretic arm and 29.9% (*p* < 0.01) for non-paretic arm. Instead, bilateral executions had significant results for rate and speed: considering FWL, there was an improvement in rate with *p* < 0.01 (31.7% for paretic arm and 37.4% for non-paretic arm), whereas speed improved by 31.2% (*p* < 0.05) and 41.7% (*p* < 0.001) for the paretic and non-paretic arm, respectively; likewise, LWL showed improvement in rate with *p* < 0.001 (29.0% for paretic arm and 27.8% for non-paretic arm) and in speed with 23.6% (*p* < 0.05) and 23.5% (*p* < 0.01) for the paretic and non-paretic arms, respectively. No significant results were recorded for gait task, although an overall good improvement was detected for arm swing asymmetry (−22.6%). Hence, this study suggests the potential benefits of continuous stimulation of upper limb function through gamified exercises and performance monitoring over medium-long periods in the home environment, thus facilitating the patient's general mobility in daily activities.

## 1 Introduction

Stroke is a clinical syndrome characterized by acute loss of focal brain function, with symptoms lasting longer than 24 h or bearing to death, caused by reduced or interrupted blood supply to a brain area (ischemic stroke) or bleeding inside brain parenchyma (hemorrhagic stroke). Despite advances in wellness, prevention, and treatment, there is an increasing incidence of stroke events in the global population, as reported by several global reports (1, 2). In addition to well-known risk factors, aging is one of the more relevant non-modifiable conditions: reports indicate that incidence doubles with age (3). The consequences of the acute event are the leading causes of various functional deficits, both in the physical and cognitive domains, resulting in a significant long-term burden on healthcare systems (4) and poor quality of life for stroke survivors (5). Indeed, stroke survivors exhibit typical motor disabilities that limit their overall mobility, directly impacting activities of daily living and active social participation (6). Specifically, hemiparesis of the contralateral upper limb is one of the most disabling manifestations: this impairment affects more than 80% of stroke survivors, causing an acute or chronic limitation of mobility, control, and coordination in the upper limbs that hinders common daily actions (e.g., reaching and picking up objects) (7). Moreover, it has been shown that the upper limbs influence gait due to the altered coordination and limited stability, being an important aspect that prevents the achievement of a normal walking speed (8).

After the acute event, specific rehabilitation protocols are promptly activated to restore lost functions, activate compensatory strategies, and improve patients' independence in daily life. Some rehabilitative therapies focus on gait, posture, and balance to reduce the risk of falls and improve patient safety (9–11). Focusing on the upper limbs, several studies pointed out how therapies based on physical exercises play a crucial role after stroke: *ad-hoc* strategies are commonly established by varying the duration, workload, and frequency according to the patient's condition and implementing dedicated training sessions based on goal-, task-, or repetition-oriented approaches (6). For example, bilateral training (i.e., exercises that stress both sides concurrently) is a recent strategy to improve motor coordination that is based on well-established knowledge. Indeed, with this approach, the non-paretic arm can stimulate the motor function of the paretic arm when simultaneous movements are performed (12).

Recently, training and rehabilitation of the upper limb through technological approaches have gained increasing interest, and various solutions have been proposed to address the severity of motor impairment in post-stroke conditions. The most widely adopted technological solutions mainly involve assistive devices (13) and robots (14, 15) exploit for the most severe conditions. Several innovative methodologies for less severe conditions include virtual reality (16, 17); serious games, exergames, and gamification techniques (14, 18); and motion tracking using vision-based systems (19–23).

In this context, we present a solution for proposing and monitoring physical activities based on gamified tasks and exercises suitable for domestic use. The primary goal is to solicit upper limb mobility through gamified tasks promoting the improvement or maintenance of upper limb motor functions, including range of motion, motor control, and coordination. The gamified tasks are offered in two modes, unilateral and bilateral execution, and can be appropriately configured for game difficulty according to subjects' motor conditions. One of the platforms implemented during the REHOME project (24) was used for the study, specifically the Motor Rehabilitation and Exergames platform (MREP) (25). MREP leverages a single RGB-Depth camera (specifically, Microsoft Azure Kinect DK) and its innovative body tracking algorithm that captures body movements in real-time through a deep learning approach. Several works have recently analyzed the performance of the device compared with gold-standard motion capture systems (MOCAP), verifying its higher accuracy compared with predecessors and other optical sensors (26–28). Other studies have also analyzed the performance of the new body-tracking algorithm, verifying its accuracy, robustness, and reliability in capturing 3D movements and poses (29–31), including the analysis of the upper limb mobility (32–34). The good agreement with MOCAPs has led to using Azure Kinect in preliminary clinical studies and rehabilitation protocols (35–38). MREP offers various exercises (grouped into assessment tasks, gamified tasks, and rehabilitative exergames) to automatically assess upper and lower limb motor impairment related to neurological disorders. However, for the purposes of this preliminary study, we included only two of the available gamified tasks and one of the assessment tasks (i.e., walking) in the experimental protocol since we intended to focus only on upper limb stimulation using gamified tasks to evaluate the potential benefits on arm swing during walking on stroke survivors, as previously done on subjects with Parkinson's disease (39). The results obtained on the cohort of stroke survivors highlight the overall improvement in upper limb mobility for both the paretic and non-paretic arms. In particular, substantial improvement in speed, number of movements per minute, coordination metrics, and reduction of asymmetry in arm swing during walking was observed, thus confirming the initial hypothesis of the potential benefits of physical activities using gamified tasks. In addition, an implicit adaptation of the performance of the non-paretic arm to the paretic arm was also observed, as in (6). It is relevant to note that these findings agree with the overall clinical improvement (scales and tests) assessed at the end of the experimental protocol, despite the specific treatment response shown by each participant. Hence, this preliminary study aimed to evaluate whether a limited number of training sessions (precisely six) with exergames could, however, contribute to improving the functionality and performance of the upper limbs in post-stroke patients over a relatively short period (2 weeks): the positive and promising results obtained from the experimental study seem to confirm this trend.

## 2 Materials and methods

### 2.1 The experimental protocol

The experimental protocol was organized in assessment and training sessions. An initial clinical assessment session (T0) was fixed to allow clinicians to assess the general motor conditions of each participant before starting the experimental protocol. Traditional scales and tests commonly used in clinical practice were selected to evaluate several motor functions: the Berg Balance Scale (BBS) (40), Trunk Impairment Test scale (TIS) (41), Time Up-and-Go test (TUG) (42), and shoulder joint mobility assessment (43). All clinical tests were administered by qualified and experienced physical therapists, following the same standardized procedure and under the same environmental conditions for all the participants to avoid bias due to the subjectivity of the assessment as much as possible. The instrumental gait motor task (G) through MREP was included in the same session to collect gait patterns and information from each participant before starting the sessions of gamified training. A final clinical and instrumental assessment session (TF) was also planned at the end of the 2-week experimental protocol (after all the gamified sessions) to compare the final motor condition to the initial one. Between T0 and TF, the training sessions using the gamified tasks offered by MREP were organized over 2 weeks. In particular, three sessions per week were planned, collecting six training sessions with gamified tasks using MREP.

A group of 11 chronic stroke subjects was recruited from the Division of Neurology and Neurorehabilitation (San Giuseppe Hospital, Istituto Auxologico Italiano, Piancavallo, Verbania, Italy), after neurological examination, according to the following inclusion criteria: mild or moderate hemiparesis with disability on the upper and lower limbs (National Institutes of Health Stroke Scale—NIHSS ≤ 10, modified Rankin Scale—mRS ≤ 3). Functional status of upper and lower limbs was also assessed considering balance status (BBS and TIS), functional ambulation (TUG), and range of mobility of the paretic shoulder (standard articular goniometer). All participants were able to walk, with or without aids, at least for short periods. There were no exclusion criteria for age, sex, side, dominance, or therapy: only cognitive impairment assessed by Mini-Mental State Examination (MMSE < 26) was considered for exclusion. The study protocol was approved by the Ethics Committee of the Istituto Auxologico Italiano IRCCS (Authorization n. 2020_02_18_01): each subject was informed about the instrumentation and experimental protocol and then provided written informed consent to participate in the study. All participants performed the experimental protocol (clinical, instrumental, and training sessions) in a supervised scenario, under the same environmental conditions, and under the supervision of the clinical staff. For this period, the activities included in the experimental protocol were performed in place of traditional rehabilitation exercises to avoid confounding variables.

### 2.2 The vision-based systems: features and tasks

As mentioned above, the MREP (25) subsystem offers numerous tasks and exergames suitable for people with neurological disorders: among them, only a specific subgroup was considered based on the primary purposes of the study, namely, to investigate the effects of gamified tasks on upper limb mobility in post-stroke subjects. The MREP consists of a vision-based system that uses a single RGB-D camera (i.e., Azure Kinect) as a non-contact sensor to collect 3D body movements in real-time.^1^ The system includes a body tracking algorithm that exploits deep learning approaches to reconstruct a 3D body skeletal model with segments and joints (44). To make interaction with the system simple and autonomous, a dedicated user interface (UI) was designed to support the user during task execution through text and audio messages. However, if necessary, the UI allows a supervisor to intervene by starting, stopping, and skipping the proposed exercises (45). A ZOTAC© ZBOX EN52060-V (16 GB RAM, NVIDIA GeForce RTX 2060 6GB, 9th generation 2.4 GHz quad-core processor) was used to run the MREP software component and manage the camera data streams. The Azure Kinect sensor was set to run at 30 fps (color and depth streams), at 1,080 p resolution (depth stream), and in Narrow Field of View (NFV) mode to detect movements from a greater distance and with a wider frontal viewing angle to prioritize tracking accuracy (27).

Regarding the experimental protocol, one instrumental evaluation task (gait) and two gamified tasks in a virtual environment were considered. In particular, the gait task was included to evaluate the potential effect of gamified tasks on arm swing ability. The gait task (G) was included to estimate subjects' ability in rhythmic arm swing and its alteration (including asymmetry) during walking, as well as some traditional spatiotemporal features of the walking pattern and trunk stability under dynamic conditions. Specifically, subjects had to complete a 6-meter-long path in front of the Azure Kinect sensor according to their best ability. Despite the short length, this path still allows the estimation of relevant gait parameters, as shown in (46).

**Figure 1:**
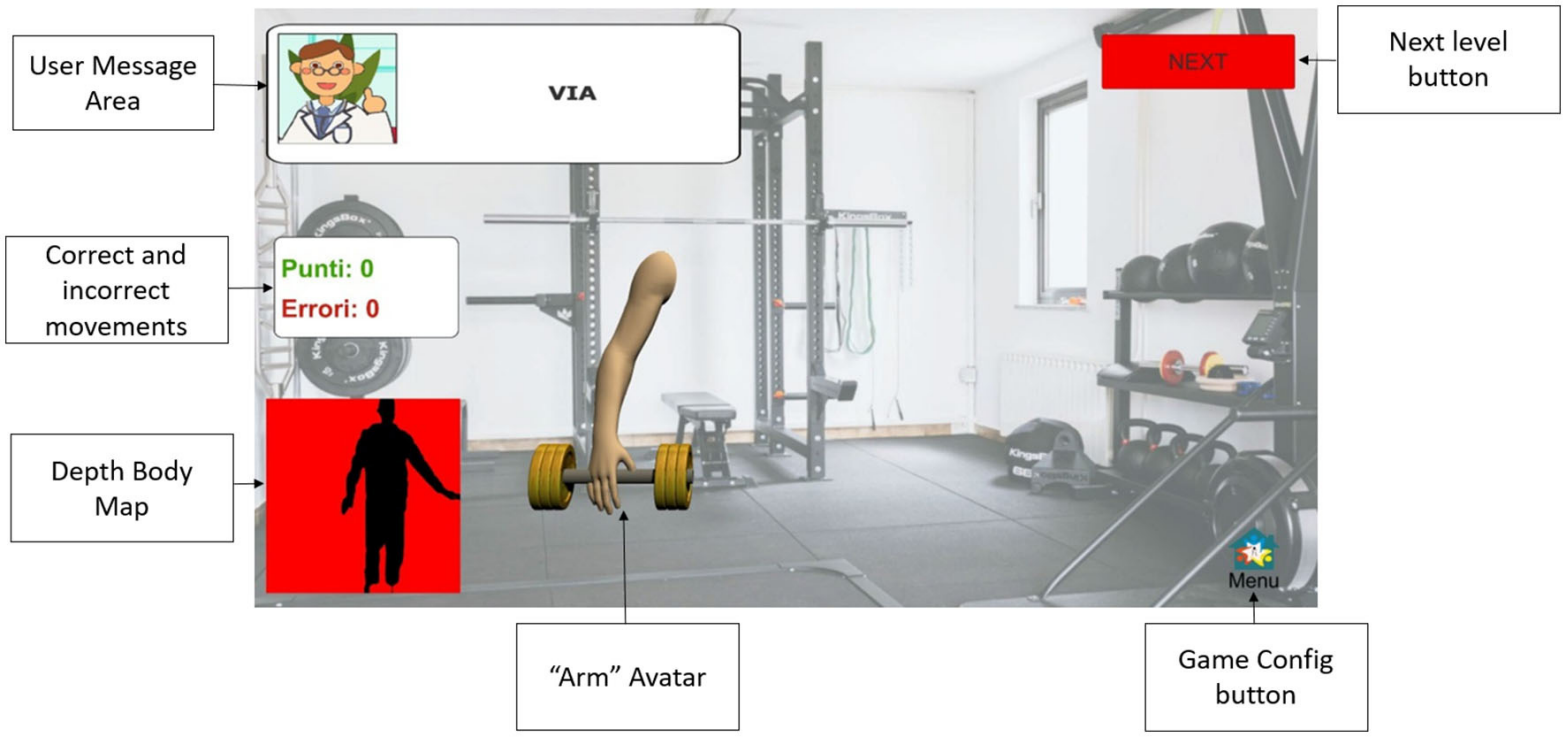
User interface for gamified tasks.

Concerning the gamified tasks, Lateral Weightlifting (LWL) and Frontal Weightlifting (FWL) games have been included (45). These tasks propose a gamified version (i.e., in a virtual environment) of exercises commonly proposed in traditional physical rehabilitation sessions to assess, train, and improve upper limb mobility, motor control, and coordination. A virtual gymnasium scenario was designed to increase subjects' engagement during exercise execution, with an avatar in the scene (i.e., arm lifting a gym weight) that reproduces the actual arm movements. Subjects had to perform, at 2.5–3 meters from the Azure Kinect sensor, a predefined number of lateral arm adduction-abduction movements (LWL) or frontal up-down movements (FWL), stressing range of motion and speed, from which some relevant mobility parameters were estimated. To stress motor control and coordination, the game exercises include unilateral (i.e., single-arm movements) and bilateral (i.e., movements of both arms simultaneously) executions. In addition, exercises can be performed in a standing or sitting position to address subjects' instability, ensure safety, and avoid the risk of falls. Finally, the exercises can be customized by setting the number of movements or the minimum arm angle threshold according to the subject's condition. Figure 1 shows a screenshot of the MREP user interface, including the description of the principal scene subareas.

The choice to include both frontal and lateral movements is because stroke survivors commonly manifest more difficulty in lateral movements (47, 48): we, therefore, expected to detect differences in the execution, control, and coordination of movements in the two proposed directions. In addition, several studies have shown that simultaneous movements stimulate the reactivation of areas in the partially damaged hemisphere, leading to improved paretic limb functions (49).

### 2.3 Estimated parameters for upper limb mobility and walking ability

Starting from the collected 3D trajectories of segments and joints of the skeletal model, task-specific functional parameters were estimated for G, LWL, and FWL using MATLAB^®^ functions and custom-written scripts.

Whole-body model acquisition from MREP allows the G task to be analyzed from three subdomains simultaneously: traditional spatiotemporal features, parameters related to lateral trunk sway (i.e., dynamic instability), and arm swing features (including asymmetry). The same approach to data analysis and feature extraction as in Ferraris et al. and Cimolin et al. (39, 46) was taken: forward and backward arm trajectories were considered to focus on the properties of arm swing, in addition to gait parameters and body stability. As noted above, instrumental gait was proposed before (T0) and after the experimental protocol (TF) to compare performance and detect differences in the three subdomains, possibly confirmed by clinical evaluation (T0 vs. TF).

**Figure 2:**
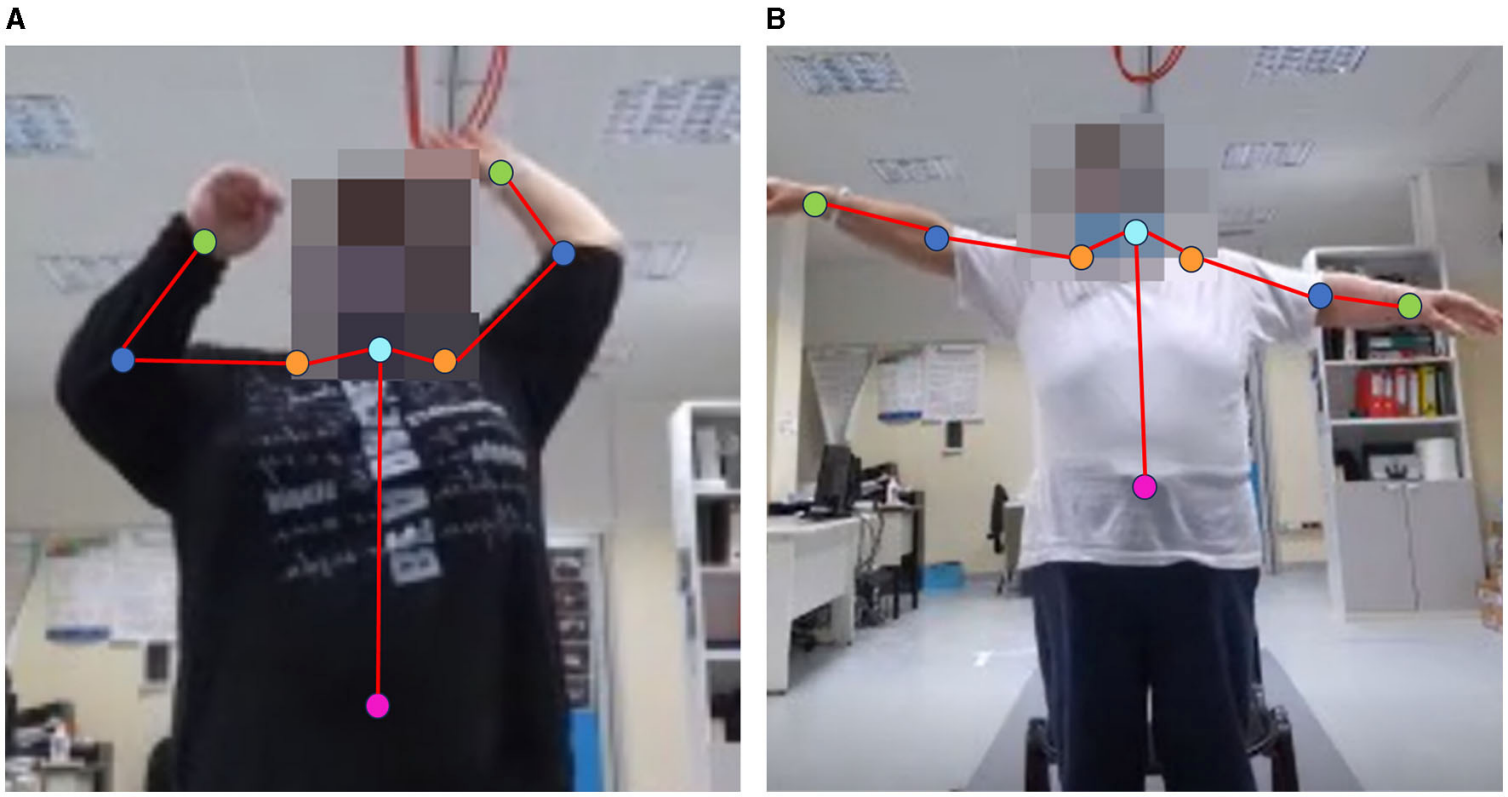
Examples of bilateral execution with skeletal model joints involved in the analysis of FWL **(A)** and LWL **(B)**: pelvis (magenta), neck (cyan), clavicles (orange), elbows (blue), wrists (green).

To analyze LWL and FWL, some joints of the skeletal model (mainly related to the upper body) were considered to estimate angular trajectories. The joints are intended to be connected in pairs to form relevant body segments for performance analysis. The body segments involved in the analysis are as follows: upper limb segment (wrist to clavicle joints); trunk segment (neck to pelvis joints); arm segment (clavicle to elbow joints); forearm segment (elbow to wrist joints). These segments defined two angular trajectories: the UPPER-LIMB-ANGLE (between upper limb and trunk segments) and the ELBOW-ANGLE (between arm and forearm segments). Since gamified tasks (LWL and FWL) required movements in different planes, the UPPER-LIMB-ANGLE was estimated with respect to the sagittal axis for the LWL (adduction-abduction movement) and to the transversal axis for the FWL (up-down movement). Figure 2 shows the joints involved in the analysis during the bilateral execution of the gamified tasks.

From the UPPER-LIMB-ANGLE trajectory, other secondary parameters such as speed and rate (i.e., number of movements per minute) were estimated, in addition to angle measurements. It should be noted that the parameters were estimated for both the paretic and non-paretic sides and for both unilateral and bilateral execution. Table 1 shows the list of the functional parameters of this study.

**Table 1:** List of parameters and metrics estimated for the study.

**Exercise**	**Parameter name**	**Meaning**	**Unit^b^**
Gait (G)	SPEED_G_	Walking speed	m/s
	${\text{STEPL}}_{\text{G}}^{\text{a}}$	Step length	m
	${\text{STANCE}}_{\text{G}}^{\text{a}}$	Duration of stance phase	% of gait cycle
	TSWAY_G_	Medio-lateral sway of trunk	mm
	${\text{ARMSW}}_{\text{G}}^{\text{a}}$	Maximum arm swing angle	deg
	ARMSYM_G_	Arm swing symmetry	–
Lateral movements (LWL)	${\text{UPANG}}_{\text{LWL}}^{\text{a}}$	Mean of maximum abduction-adduction movements angle	deg
	${\text{ELANG}}_{\text{LWL}}^{\text{a}}$	Mean elbow angle	deg
	${\text{SPEED}}_{\text{LWL}}^{\text{a}}$	Mean speed of lateral movements	deg/s
	${\text{RATE}}_{\text{LWL}}^{\text{a}}$	Lateral movements per minute	mov/min
	SYNC_LWL_	Synchronicity index (bilateral execution only)	–
	SIMIL_LWL_	Similarity index (bilateral execution only)	–
Frontal movements (FWL)	${\text{UPANG}}_{\text{FWL}}^{\text{a}}$	Mean of maximum up-down movements angle	deg
	${\text{ELANG}}_{\text{FWL}}^{\text{a}}$	Mean elbow extension angle	deg
	${\text{SPEED}}_{\text{FWL}}^{\text{a}}$	Mean speed of frontal movements	deg/s
	${\text{RATE}}_{\text{FWL}}^{\text{a}}$	Frontal movements per minute	mov/min
	SYNC_FWL_	Synchronicity index (bilateral execution only)	–
	SIMIL_FWL_	Similarity index (bilateral execution only)	–

^a^Parameters estimated separately for the paretic and the non-paretic side.

^b^Symbol—indicates numerical parameter without unit.

In addition to the more traditional measurements (angles, speeds, and related measures), three metrics were considered for a more in-depth view of arm mobility, especially during simultaneous movements: ARMSYM_G_ for the gait task, SYNC and SIMIL for the LWL and FWL tasks. ARMSYM_G_ is an index calculated as in (39) to assess arm swing asymmetry during gait: more severe asymmetry (considering maximum forward and backward arm angles) corresponds to more negative ARMSYM_G_ values. Therefore, a lower negative index value indicates an improvement in arm swing asymmetry.

In order to emphasize differences between upper limb trajectories during simultaneous movements (i.e., bilateral execution), SYNC and SIMIL metrics were included to provide a summary measure devoted explicitly to the temporal and spatial symmetry of bilateral movements, thus gaining insights into motor control and coordination.

In particular, the SYNC metric (45) refers to the temporal synchronization of simultaneous arm movements by quantifying the time lag that occurs between the upper limb trajectories above and below the preset minimum angular threshold and the consequent correspondence in bilateral movement cycles: values closer to zero are associated with good temporal synchronization during bilateral movements; values farther from zero indicate unsynchronized movements. Therefore, an improvement in temporal coordination is indicated by decreasing values of this metric.

**Figure 3:**
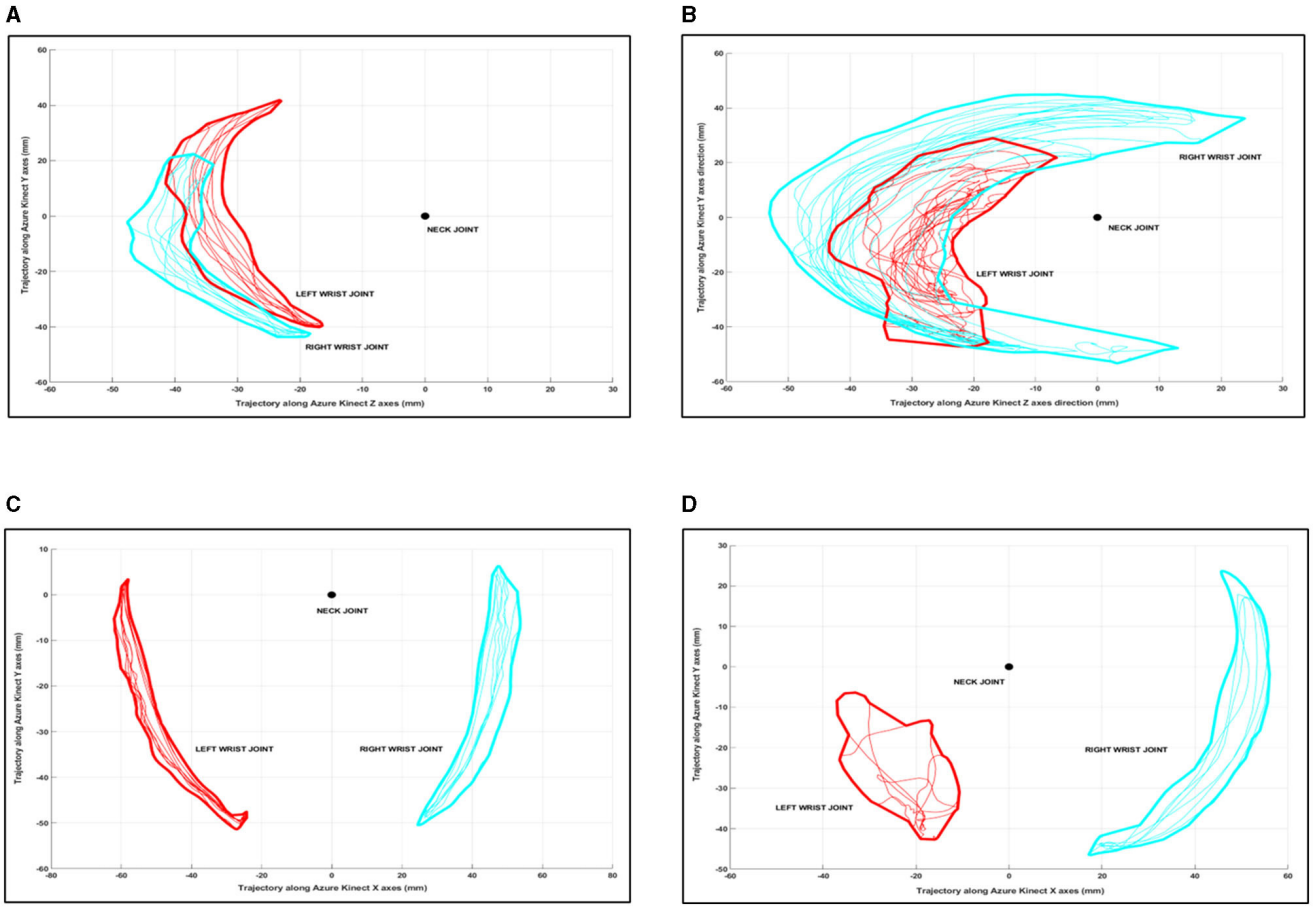
Examples of shape similarity during gamified tasks: good shape similarity in FWL **(A)**, poor shape similarity in FWL **(B)**, good shape similarity in LWL **(C)**, and poor shape similarity in LWL **(D)**.

The SIMIL metric evaluates the similarity between the 2D shapes drawn by the WRIST joint trajectories with respect to a common reference point (i.e., the NECK joint) in the two main directions of motion (according to lateral and frontal movements). To estimate the SIMIL metric, the MATLAB *procrustes* function (with scaling parameter disabled) was used to obtain information about the different characteristics of the paretic and non-paretic arm trajectories during bilateral movements. The *procrustes* function returns values close to zero for shapes with good similarity and increasing values for shapes with poor similarity. Examples of shapes drawn during bilateral execution are shown in Figure 3.

As mentioned above, LWL and FWL tasks were proposed during the six training sessions (R1-R6) of the 2-week experimental protocol: parameters and metrics were estimated for each session. In addition, to detect performance improvements and trends, they were averaged and then compared for the first and the second week.

### 2.4 Statistical analysis

Statistical analysis was performed using Jamovi (version 2.2.5), an open-source modular platform for statistical computing (50), considering a 95% significance level (*p* < 0.05) for statistical tests. Considering the relatively small number of subjects and sessions, we tested the normality distribution of the estimated parameters through the Shapiro-Wilk test. Then, the distribution of estimated features was compared using parametric or non-parametric tests for paired samples to support our results with statistical evidence. Since all the considered parameters and clinical data showed normal distribution, data were provided as mean and standard deviation, while parametric tests (*t*-test) were used for statistical analysis.

## 3 Results

### 3.1 Clinical outcomes

A total of 11 post-stroke volunteers were deemed eligible and included in this single cohort study; however, one subject withdrew after the second gamified session due to personal reasons not related to difficulties with the experimental protocol, and thus was excluded from the subsequent analysis. Table 2 shows the demographic characteristics of the 10 participants who correctly completed the experimental protocol.

**Table 2:** Participants' characteristics: demographic and clinical data (T0).

**Participants' characteristics**	**Value**
Number (#)	10
Average age (years)	72.0 ± 10.5
Gender (#)	8 males/2 females
Years from acute event (years)	6.3 ± 5.1
Paretic side (#)	3 left/7 right

All the participants were able to walk (during task G) without assistive devices (such as tripods or canes), although five of the 10 participants routinely used them. Hence, a sitting position was preferred for the same subjects to ensure safety during the gamified tasks. The instrumental and training sessions were correctly completed by all subjects, except one subject who was unable to perform bilateral execution in most of the scheduled training sessions and one subject who needed to be supported by the supervisor during ambulation without assistive devices: the corresponding data were then discarded, resulting in 54 trials included for the analysis of LWL and FWL, and 18 trials included for the gait analysis.

Data analysis revealed an overall improvement in motor performance at the end of the experimental protocol (TF) for all clinical metrics considered. Specifically, TIS and BERG scores increased, as did paretic shoulder mobility angles, while TUG time decreased: all these changes denote an average improvement in patients' performance in clinical assessment of specific domains. However, only TUG and BERG show a statistically significant difference (*p* < 0.05), while TIS and paretic shoulder mobility are near significance (*p* < 0.06). The average improvement in clinical data and the percentage change at the end of the experimental protocol are shown in Table 3. These results suggest a trend of general improvement in motor condition, although each participant showed a different response to the protocol, as indicated by Table 4.

**Table 3:** Average and percentage improvement of clinical assessment (TF vs. T0) over all participants.

**Clinical assessment**	**T0**	**TF**	**TF vs. T0 (%)**
Berg scale score (points)	36.6 ± 15.7	40.2 ± 15.9	+9.8%^*^
TIS scale score (points)	11.0 ± 3.8	13.3 ± 4.3	+20.9%
TUG test (seconds)	28.4 ± 15.8	24.9 ± 15.4	−12.3%^*^
Paretic shoulder mobility (degree)	131.0 ± 31.8	146.0 ± 28.4	+11.5%

^*^p < 0.05.

**Table 4:** Percentage change in clinical assessment (TF vs. T0) of each participant.

**#ID^a^**	**TIS (%)^b^**	**TUG (%)^b^**	**BERG (%)^b^**	**Shoulder mobility (%)^b^**
#PT1	+38.5%	−27.8%	+10.0%	–
#PT3	–	–	–	–
#PT4	+77.8%	−1.5%	−16.7%	+6.3%
#PT5	–	−5.6%	+16.7%	+8.3%
#PT6	+26.7%	+12.5%	+1.9%	–
#PT7	+33.3%	−11.1%	–	+14.3%
#PT8	+66.7%	−27.4%	+52.0%	+66.7%
#PT9	–	−29.1%	+3.6%	–
#PT10	–	−18.1%	+21.6%	+33.3%
#PT11	–	−10.5%	–	+9.1%

^a^#PT2 was excluded from the analysis (withdrew after the second gamified session).

^b^Symbol—indicates no percentage change in clinical assessment.

### 3.2 Gait task: main results

This subsection is devoted to showing differences in the intergroup walking ability at the end of the experimental protocol. The average estimated gait parameters for T0 and TF (Table 1) and their percentage changes are shown in Table 5.

**Table 5:** Mean values (with standard deviation) and percentage changes of gait parameters (TF vs. T0) over all participants.

**Parameter**	**T0**	**TF**	**TF vs. T0 (%)**
STEPL_G_ (m)^a^	0.36 ± 0.15	0.35 ± 0.13	−2.9%
SPEED_G_ (m/s)	0.50 ± 0.25	0.45 ± 0.23	−8.5%
STANCE_G_ (% of gait cycle)^a^	77.00 ± 12.10	76.62 ± 8.36	−0.4%
TSWAY_G_ (mm)	108.00 ± 20.00	111.07 ± 36.54	+2.9%
ARMSW_G_ (deg)^a^	40.63 ± 19.35	33.73 ± 20.93	−16.5%
ARMSYM_G_ (-)	−16.31 ± 13.70	−12.54 ± 9.76	−22.6%

^a^Parameters estimated as mean value of the paretic and the non-paretic side.

As reported in Table 5, almost all parameters show a relatively stable trend. Walking speed and step length show negligible intergroup deterioration, as does dynamic stability (approximately −0.05 m/s, −0.01 m, and +4 mm, respectively). In contrast, the stance phase duration slightly decreased (i.e., improved) in the gait cycle (−0.3%). Focusing on the arm swing, the maximum angle (averaged over both arms) slightly deteriorated (about −7.0 degrees). However, the most interesting result concerns the asymmetry index: ARMSYM_G_ shows a substantial reduction in its negative value at TF, suggesting an improvement in arm swing ability and motor coordination despite lower absolute arm angles. However, the statistical analysis found no significant difference (*p* > 0.05) for all estimated gait parameters, including ARMSYM_G_: this could be due to each subject's different response to the protocol (as occurs for clinical assessment) or the need for a longer protocol duration to obtain statistical evidence of overall improvement in fine-grained gait parameters.

### 3.3 LWL and FWL tasks: main results of unilateral execution

This subsection is devoted to showing intergroup differences and trends for the LWL and FWL tasks by comparing the average parameters (Table 1) estimated for the first and second weeks of the experimental protocol. The estimated parameters for the 2 weeks and the percentage changes for the paretic and non-paretic sides are shown in Table 6.

**Table 6:** Unilateral execution: trends of parameters for LWL and FWL (paretic arm and non-paretic arm) over all participants.

**Exercise**	**Parameter**	**Week 1**	**Week 2**	**Week 2 vs. week 1 (%)**
Frontal movements (FWL)		**Paretic arm**		
	UPANG_FWL_ (deg)	103.03 ± 22.47	103.00 ± 20.38	−0.1%
	ELANG_FWL_ (deg)	123.18 ± 17.42	122.57 ± 17.78	−0.5%
	SPEED_FWL_ (deg/s)	62.04 ± 24.61	79.16 ± 36.20	+27.6%
	RATE_FWL_ (mov/min)	16.14 ± 5.77	22.35 ± 6.12	+38.5% (^***^)
		**Non-paretic arm**		
	UPANG_FWL_ (deg)	124.60 ± 24.60	125.84 ± 26.86	+1.0%
	ELANG_FWL_ (deg)	138.50 ± 10.95	138.14 ± 11.82	−0.3%
	SPEED_FWL_ (deg/s)	78.90 ± 31.33	89.41 ± 37.67	+13.4%
	RATE_FWL_ (mov/min)	15.97 ± 4.33	21.54 ± 4.83	+34.9% (^**^)
Lateral movements (LWL)		**Paretic arm**		
	UPANG_LWL_ (deg)	91.45 ± 20.21	93.04 ± 23.85	+1.7%
	ELANG_LWL_ (deg)	125.87 ± 18.73	128.61 ± 15.33	+2.2%
	SPEED_LWL_ (deg/s)	61.71 ± 25.24	72.88 ± 40.93	+18.1%
	RATE_LWL_ (mov/min)	18.88 ± 6.38	22.64 ± 6.89	+19.9% (^*^)
		**Non-paretic arm**		
	UPANG_LWL_ (deg)	117.41 ± 21.62	119.60 ± 28.53	+1.9%
	ELANG_LWL_ (deg)	145.81 ± 6.34	142.39 ± 9.88	−2.4%
	SPEED_LWL_ (deg/s)	79.82 ± 20.12	90.08 ± 45.98	+12.8%
	RATE_LWL_ (mov/min)	19.38 ± 4.40	25.18 ± 5.47	+29.9% (^**^)

^*^p < 0.05; ^**^p < 0.01; ^***^p < 0.001.

First, the gamified tasks highlight a significant difference between paretic and non-paretic arm performance, as expected (lower performance for the paretic arm). More specifically, while the angular parameters (i.e., UPPER-LIMB-ANGLE and ELBOW-ANGLE) show irrelevant changes in the second week (*p* > 0.05), an improvement in speed and rate is substantial for both tasks as confirmed by the statistical analysis for rate (*p* < 0.05). Other insights emerge from the analysis. Frontal movements (UPANG_FWL_) seem to promote higher upper limb angles than lateral movements (UPANG_LWL_), confirming the greater difficulty of post-stroke subjects in controlling lateral movements. In contrast, lateral movements seem to favor the proper extension of the upper limbs during the exercises, as indicated by elbow angles (ELANG_LWL_ > ELANG_FWL_). As concluding remark, the frontal movements seem to promote more noticeable improvements on the paretic limb compared to lateral movements, although significant changes in speed and rate have still been observed in both.

### 3.4 LWL and FWL tasks: main results of bilateral execution

This subsection aims to show intergroup differences and trends for the bilateral execution of LWL and FWL tasks by comparing the average parameters (Table 1) estimated for the first and second weeks of the experimental protocol. The estimated parameters for the 2 weeks and the percentage changes for the paretic and non-paretic sides are shown in Table 7.

**Table 7:** Bilateral execution: trends of parameters for LWL and FWL (paretic arm and non-paretic arm) over all participants.

**Exercise**	**Parameter**	**Week 1**	**Week 2**	**Week 2 vs. week 1 (%)**
Frontal movements (FWL)		**Paretic arm**		
	UPANG_FWL_ (deg)	105.08 ± 21.25	110.22 ± 23.84	+4.9%
	ELANG_FWL_ (deg)	128.57 ± 17.03	128.31 ± 16.37	−0.3%
	SPEED_FWL_ (deg/s)	62.64 ± 29.11	82.19 ± 35.69	+31.2% (^*^)
	RATE_FWL_ (mov/min)	15.50 ± 5.48	21.34 ± 6.56	+37.7% (^**^)
		**Non-paretic arm**		
	UPANG_FWL_ (deg)	118.16 ± 23.78	119.36 ± 26.23	+1.0%
	ELANG_FWL_ (deg)	137.95 ± 14.51	135.23 ± 17.71	−2.0%
	SPEED_FWL_ (deg/s)	61.46 ± 24.84	87.07 ± 28.15	+41.7% (^**^)
	RATE_FWL_ (mov/min)	15.67 ± 5.48	21.53 ± 6.34	+37.4% (^**^)
Lateral movements (LWL)		**Paretic arm**		
	UPANG_LWL_ (deg)	85.45 ± 22.66	82.06 ± 22.47	−4.0%
	ELANG_LWL_ (deg)	125.04 ± 18.54	129.13 ± 12.46	+3.3%
	SPEED_LWL_ (deg/s)	50.83 ± 23.59	62.83 ± 28.58	+23.6% (^*^)
	RATE_LWL_ (mov/min)	17.53 ± 7.73	22.62 ± 6.68	+29.0% (^***^)
		**Non-paretic arm**		
	UPANG_LWL_ (deg)	109.14 ± 15.69	106.56 ± 19.15	−2.4%
	ELANG_LWL_ (deg)	139.79 ± 6.05	140.92 ± 5.59	+0.8%
	SPEED_LWL_ (deg/s)	68.86 ± 26.50	85.02 ± 29.98	+23.5% (^**^)
	RATE_LWL_ (mov/min)	17.90 ± 7.47	22.87 ± 6.83	+27.8% (^***^)

^*^p < 0.05; ^**^p < 0.01; ^***^p < 0.001.

As with the unilateral execution, the bilateral performance in LWL and FWL confirms the previous results, with negligible differences for angular measures (*p* > 0.05) but substantial improvement in speed and rate (*p* < 0.05). The detected improvement is a very relevant result, as it was obtained during a more complex exercise requiring more motor control and coordination. Other insights emerge from the analysis of the SYNC and SIMIL metrics (Table 8). Regarding the FWL, the time synchronization (SYNC_FWL_) of both arms improves significantly in the second week (*p* < 0.05), while the shape similarity shows no relevant changes. In contrast, LWL shows a minimal but not significant deterioration in both metrics. It is important to note that the SYNC metric is relatively low (< 0.4) for both tasks, denoting good movement synchronization in time for the group of participants. The value is also low for the SIMIL metric in the FWL task. At the same time, it is slightly higher for the LWL task, confirming that post-stroke subjects have more difficulty in spatial coordination of lateral movements (SIMIL_LWL_ > SIMIL_FWL_).

**Table 8:** Bilateral execution: trends of mean metrics (with min-max range) for LWL and FWL over all participants.

**Exercise**	**Metric**	**Week 1**	**Week 2**	**Week 2 vs. week 1 (%)**
Frontal movements (FWL)	SYNC_FWL_	0.27 (0.03–0.91)	0.14 (0.01–0.63)	−49.2% (^*^)
	SIMIL_FWL_	0.21 (0.01–0.96)	0.21 (0.01–1.05)	−0.2%
Lateral movements (LWL)	SYNC_LWL_	0.28 (0.03–1.13)	0.31 (0.02–0.96)	+7.5%
	SIMIL_LWL_	0.98 (0.01–6.82)	1.03 (0.01–6.22)	+4.6%

^*^p < 0.05.

The last result comes from comparing upper limb performance during unilateral and bilateral execution (Table 9). The analysis shows that the maximum UPPER-LIMB-ANGLE (UPANG_FWL_ and UPANG_LWL_ parameters) is lower during bilateral than unilateral execution, except for frontal movements of the paretic arm. The same is valid for rate parameters (RATE_FWL_ and RATE_LWL_), where performance in bilateral execution is always lower. This outcome suggests an implicit adaptation of the non-paretic arm to the limited capability of the paretic one in terms of movement amplitude and velocity. However, the most significant differences are found during lateral movements regarding maximum lift angle and frequency of movements.

**Table 9:** Comparison between unilateral (UNI) and bilateral (BI) execution for the upper limb angle and rate (number of movements per minute) parameters over all participants.

**Exercise**	**Week**	**UNI**	**BI**	**Diff (%)**	**UNI**	**BI**	**Diff (%)**
Frontal movements (FWL)		**UPANG (paretic)**			**RATE (paretic)**		
	1	103.03	105.08	+2.0%	16.14	15.50	−4.0%
	2	103.00	110.22	+7.0%	22.35	21.34	−4.5%
		**UPANG (non paretic)**			**RATE (non paretic)**		
	1	124.60	118.16	−5.2%	15.97	15.67	−1.9%
	2	125.84	119.36	−5.2%	21.54	21.53	−0.1%
		**UPANG (paretic)**			**RATE (paretic)**		
Lateral movements (LWL)	1	91.45	85.45	−6.6%^*^	18.88	17.53	−7.2%^*^
	2	93.04	82.06	−11.8%^*^	22.64	22.62	−0.1%
		**UPANG (non paretic)**			**RATE (non paretic)**		
	1	117.41	109.14	−7.0%^*^	19.38	17.90	−7.6%
	2	119.6	106.56	−10.9%^*^	25.18	22.87	−9.2%^*^

^*^p < 0.05.

## 4 Discussion

This preliminary study assessed the exergames as an easy-to-use and engaging tool to enhance upper limb mobility in post-stroke subjects in a 2-week experimental protocol that included six training sessions. The results showed an overall improvement for several motor functions measured with scales and tests, such as shoulder joint mobility, posture (TIS scale), balance (BERG scale), and walking (TUG test). The results of the functional parameters support these achievements; in fact, we had significant improvements for both the frontal and lateral execution performed unilaterally and bilaterally with an increase of speed and rate (i.e., number of movements per minute) for both the paretic and non-paretic side, suggesting that an extended treatment could improve the upper limb mobility with positive influence also on trunk control and balance (51). Additionally, during the second week, there was an improvement in the synchronization metric for FWL, probably due to the neural plasticity process (52). However, it did not occur for LWL, probably due to the more difficult motor coordination during lateral execution. Then, the comparison of unilateral and bilateral executions showed that the bilateral execution had a smaller maximum angle for all the examined conditions apart from the paretic arm in FWL, highlighting the significant complexity characterizing the execution and control of the simultaneous movements. Finally, gait remained substantially stable, showing interesting changes only for the arm swing during walking: the reduction of both the maximum swing angle and the arm swing asymmetry suggests greater coordination in arm swing movements despite the lower amplitude. Instead, the lack of tangible improvement of the other gait parameters is justifiable by their nature: they are all related to the lower limbs, while the gamified tasks of this experimental protocol stimulate only the upper limb mobility. Hence, *ad-hoc* gamified tasks for the trunk, lower limbs, and balance should be created and tested as well.

Our preliminary results seem to confirm a positive trend for all participants in upper limb motor performance, even in bilateral execution, suggesting that prolonged treatment could produce many benefits for upper limb control and coordination, with consequent positive effects on overall motor condition and quality of life: by stimulating the strength, neuromuscular aspects and both paretic and non-paretic arms, the exergames improve the patients' autonomy, allowing them to maintain the functionality of those movements that confer independence, such as the personal hygiene.

The gamified tasks included frontal and lateral lifting movements, joined with unilateral and bilateral execution, to stimulate the upper limb motor function and mobility differently. This choice additionally allowed the solicitation of both motor control and coordination. Moreover, these gamified tasks also solicit the cognitive aspect: as shown in this study, exergames do not have a static difficulty. Instead, they allow the game level to be changed according to the patient's needs and characteristics: this means game (i.e., task) difficulty can be reconfigured based on the actual motor condition. If motor function improves, the game task can be configured to a superior difficulty. In contrast, difficulty can be reduced if the task is perceived as too complicated for the patient's status. Consequently, the patients become the “players” of this individualized and constantly stimulating rehabilitation therapy whose virtual environment and playful real-world scenario make Exergames suitable and ideal for the home setting: people with motor and cognitive deficits related to neurological disorders could continue the rehabilitation program at home, repeating the exercises not to forget the previously re-learned tasks. From an economic point of view, this home rehabilitation option would reduce healthcare costs and provide a helpful rehabilitation strategy that is also suitable for poor areas.

Specifically, post-stroke is one of the neurological impairments that could benefit from this type of solution. Stroke survivors promptly undergo in-hospital rehabilitation after the acute phase to begin recovery of motor functions impaired by the event as soon as possible. Despite this, most patients would need continuous and frequent maintenance activities to avoid losing the functional recovery achieved. However, this is not feasible in a hospital setting and is cost-demanding in an outpatient setting. Telemonitoring and telerehabilitation solutions could fill this gap, and exergames could prove to be efficient in ensuring continuity of treatment, facilitating the execution of specific physical exercises, stimulating the achievement of new rehabilitation goals, and ensuring greater adherence to treatment through a fun and engaging approach between maintenance rehabilitation sessions. Furthermore, the 3D body tracking system provides an easy-to-use and non-invasive way to collect patient mobility and performance data, allowing extensive monitoring, even during the off-period of annual rehabilitation cycles, which is also suitable for home settings. Hence, with a constant monitoring process, the scheduling of rehabilitation cycles may be improved, thus becoming more cost-effective and tailored to patients' needs.

This preliminary study is not without limitations. First, we have a limited number of subjects and sessions performed to draw generalized conclusions. It will be necessary to involve more subjects and to consider a longer observation period compatible with the duration of traditional rehabilitation protocols to generalize and consolidate current results. Then, a comparison with healthy subjects undergoing the same protocol based on gamified tasks is currently lacking: a future study will be organized to evaluate differences with a control group. In addition, to evaluate the effectiveness of the proposed gaming protocol, it will also be necessary to organize a dedicated and more extensive clinical study comparing post-stroke subjects undergoing only the gaming protocol and others undergoing concurrent upper limb rehabilitation treatments, although this study will necessarily require longer times to be completed. However, it will be necessary to consider a more prolonged double-blinded or cross-over study with larger sample for comparing clinical efficacy of the game-based protocol. Finally, as mentioned above, some instrumental measures showed an improvement trend in line with the clinical evaluation at the end of the gamified protocol (TF). Although clinical scales potentially vulnerable to rater subjectivity were used, the clinical evaluation was performed by qualified and experienced personnel, following standardized procedures and under the same conditions for all participants that should have mitigated the potential bias in the results due to this factor. Nevertheless, further studies will be necessary to consolidate what was observed in this study.

In conclusion, the encouraging data obtained in this study promotes the implementation of this technology, especially for monitoring and training/maintenance of motor functions in the domestic environment. Despite 2 weeks of training sessions are few in terms of rehabilitation, the problem-solving and visuospatial transformations typical of the gamified exercises have demonstrated fascinating potential. By combining the neurophysiological basis of rehabilitation in stroke patients with the potential of technological solutions, the system we studied may maintain, and perhaps improve, the gesture functionality acquired with intensive rehabilitation. Moreover, the patient's remote monitoring, the activities of daily living maintenance, and cognitive engagement may contribute to reducing the costs of the National Health Service and promoting new rehabilitation solutions in low-income countries. Notwithstanding this, it will be necessary to extend the analysis to a larger group of subjects, not necessarily post-stroke, with a more extended study time to investigate the effectiveness of the proposed solution.

As future developments, we will evaluate the possibility of automatically configuring gamified tasks through artificial intelligence (AI) models that weigh the subjects' functional capabilities and motor performance to adjust game levels appropriately: on the one hand, AI models could contribute to avoiding emotional stress (anxiety, distrust, demoralization), but on the other, they could set a proper and optimized challenging level for stimulating patients' constant improvement. In addition, we are also planning the integration of new gamified tasks dedicated to hand dexterity to comprehensively enhance and stimulate the motor functions related to the upper limbs.

## Data availability statement

The datasets presented in this study can be found in online repositories. The name of the repository and accession number can be found below: Zenodo, https://zenodo.org/, DOI: 10.5281/zenodo.10207753.

## Ethics statement

The studies involving humans were approved by Ethics Committee of Istituto Auxologico Italiano IRCCS (Authorization n. 2020_02_18_01). The studies were conducted in accordance with the local legislation and institutional requirements. The participants provided their written informed consent to participate in this study.

## Author contributions

LV: Conceptualization, Data curation, Formal analysis, Investigation, Methodology, Visualization, Writing – original draft, Writing – review & editing. CF: Conceptualization, Data curation, Formal analysis, Investigation, Methodology, Supervision, Visualization, Writing – original draft, Writing – review & editing. GA: Data curation, Investigation, Methodology, Software, Validation, Writing – review & editing. GP: Data curation, Project administration, Resources, Writing – review & editing. FB: Writing – original draft, Writing – review & editing. AT: Methodology, Writing – original draft, Writing – review & editing. AM: Funding acquisition, Project administration, Resources, Supervision, Writing – review & editing. LP: Formal analysis, Investigation, Methodology, Supervision, Writing – review & editing.

## Footnotes

^1^Azure Kinect DK. Available from: https://azure.microsoft.com/it-it/products/kinect-dk/ (accessed November 10, 2023).
